# Longitudinal association between inflammatory markers and specific symptoms of depression in a prospective birth cohort

**DOI:** 10.1016/j.bbi.2018.11.007

**Published:** 2019-02

**Authors:** Alexander L. Chu, Jan Stochl, Glyn Lewis, Stan Zammit, Peter B. Jones, Golam M. Khandaker

**Affiliations:** a Department of Psychiatry, School of Clinical Medicine, University of Cambridge, Cambridge, UK; b Department of Kinanthropology, Charles University, Prague, Czech Republic; c Division of Psychiatry, University College London, London, UK; d Centre for Academic Mental Health, Population Health Sciences, Bristol Medical School, University of Bristol, Bristol, UK; e Institute of Psychological Medicine and Clinical Neurosciences, Cardiff University, Cardiff, UK; f Cambridgeshire and Peterborough NHS Foundation Trust, Cambridge, UK

**Keywords:** ALSPAC, Avon Longitudinal Study of Parents and Children, BMI, body mass index, CI, confidence interval, CIS-R, Clinical Interview Schedule-Revised, CRP, C-reactive protein, ELISA, enzyme-linked immunosorbent assay, ICD-10, International Classification of Diseases 10th Revision, IL-6, interleukin 6, RR, risk ratio, SD, Standard Deviation, SEM, structural equation modelling, Depression, Psychological Symptoms, Somatic symptoms, Inflammation, Interleukin 6, C-reactive protein, Cohort study, Immunopsychiatry, ALSPAC, Neurovegetative Symptoms

## Abstract

**Background:**

Low-grade inflammation is associated with depression, but studies of specific symptoms are relatively scarce. Association between inflammatory markers and specific symptoms may provide insights into potential mechanism of inflammation-related depression. Using longitudinal data, we have tested whether childhood serum interleukin 6 (IL-6) and C-reactive protein (CRP) levels are associated with specific depressive symptoms in early adulthood.

**Methods:**

In the ALSPAC birth cohort, serum IL-6 and CRP levels were assessed at age 9 years and 19 depressive symptoms were assessed at age 18 years. We used modified Poisson generalised linear regression with robust error variance to estimate the risk ratio (RR) and 95% confidence interval (95% CI) for each depressive symptom. In addition, we used confirmatory factor analysis to create two continuous latent variables representing somatic/neurovegetative and psychological dimension scores. Structural equation modelling was used to test the associations between IL-6 and these dimension scores.

**Results:**

Based on data from 2731 participants, IL-6 was associated with diurnal mood variation, concentration difficulties, fatigue and sleep disturbances. The adjusted RRs for these symptoms at age 18 years for participants in top, compared with bottom, third of IL-6 at age 9 years were 1.75 (95% CI, 1.13–2.69) for diurnal mood variation, 1.50 (95% CI, 1.11–2.02) for concentration difficulties, 1.31 (95% CI, 1.12–1.54) for fatigue, and 1.24 (95% CI, 1.01–1.52) for sleep disturbances. At dimension level, IL-6 was associated with both somatic/neurovegetative (β = 0.059, SE = 0.024, *P* = 0.013) and psychological (β = 0.056, SE = 0.023, *P* = 0.016) scores.

**Conclusions:**

Inflammation is associated with specific symptoms of depression. Associations with so-called somatic/neurovegetative symptoms of depression such as fatigue, sleep disturbances and diurnal mood variation indicate that these symptoms could be useful treatment targets and markers of treatment response in clinical trials of anti-inflammatory treatment for depression.

## Highlights

• At symptom level, IL-6 is associated with diurnal variation in mood, concentration difficulties, fatigue and sleep disturbances.
• These are so-called somatic/neurovegetative symptoms of depression.
• At dimension level, IL-6 is associated with both somatic/neurovegetative and psychological symptom dimensions.
• Somatic/neurovegetative symptoms could be useful treatment targets and markers of treatment response in clinical trials of anti-inflammatory treatment for depression.

## 1 Introduction

An association between low-grade inflammation and depression is well established. Meta-analyses of cross-sectional studies confirm that serum concentrations of pro-inflammatory cytokines [e.g., interleukin 6 (IL-6)] and acute phase proteins [e.g., C-reactive protein (CRP)] are elevated in acutely unwell patients with depression in comparison with controls (Dowlati et al., 2010, Goldsmith et al., 2016, Haapakoski et al., 2015, Howren et al., 2009). These elevated levels tend to normalise after recovery upon treatment with antidepressants, but continue to remain elevated in treatment resistant patients (Goldsmith et al., 2016, Maes et al., 1997, O'Brien et al., 2007). Longitudinal studies reporting an association between higher levels of IL-6 and CRP in childhood/adulthood and subsequent development (Gimeno et al., 2009, Khandaker et al., 2014) and persistence (Khandaker et al., 2017b, Zalli et al., 2016) of depressive symptoms indicate that inflammation could be a cause, rather than simply a consequence of the illness.

Depression consists of a diverse range of symptoms. Inflammation may contribute to pathogenesis of specific components of the syndrome. Symptoms such as fatigue, psychomotor retardation, impaired sleep and change in appetite are often referred to as sickness behavior or neurovegetative symptoms in the experimental animal literature (Dantzer et al., 2008, Miller et al., 2009, Raison et al., 2006). The International Classification of Diseases 10th Revision (ICD-10) diagnostic criteria for depressive episode specifies these symptoms along with a few others as somatic symptoms of depression (see below) (WHO, 1992). Somatic symptoms are different from psychological symptoms (e.g., hopelessness and guilt) of depression phenomenologically (WHO, 1992) and perhaps physiologically (Jokela et al., 2016). Cross-sectional studies indicate that low-grade inflammation, as measured by elevated serum CRP levels, is associated with somatic symptoms (e.g., fatigue, impaired sleep and activity levels) but not with psychological symptoms within the general population or in depressed patients (Jokela et al., 2016, Kohler-Forsberg et al., 2017). However, these studies did not compare effect estimates for somatic and psychological dimensions directly. Atypical features of depression, particularly increased appetite or weight, are associated with a number of genetic risk variants for body mass index (BMI) and levels of CRP and leptin (Milaneschi et al., 2017). These findings support the idea that different depressive symptoms may have distinct pathophysiological mechanisms. Association between markers of inflammation and specific symptoms may provide important clues regarding potential mechanisms of inflammation-related depression. However, longitudinal studies of inflammatory markers and specific symptoms of depression are scarce.

Using data from the general population-representative Avon Longitudinal Study of Parents and Children (ALSPAC) birth cohort, we previously reported a longitudinal association between serum IL-6 levels in childhood at age 9 years and risk of depression and psychosis subsequently in early adulthood at age 18 years (Khandaker et al., 2014). In order to gain further insight into the relationship between inflammation and depression, we present another longitudinal study based on the ALSPAC cohort that examines the relationships between IL-6 and CRP at age 9 years and 19 specific symptoms of depression assessed at age 18 years. In addition to using individual depressive symptoms, we created two latent variables representing somatic/neurovegetative and psychological symptom dimension scores. We hypothesised that childhood inflammatory markers would be associated with specific depressive symptoms in early adulthood.

## 2 Methods

### 2.1 Description of cohort and sample selection

ALSPAC is a general population-based birth cohort in the former Avon County in the South West region of England. 14,541 pregnant women who were residents of the study catchment areas and had expected delivery dates between April 1st, 1991 and December 31st, 1992 were initially recruited into the cohort, resulting in 14,062 live births. Additional children and parents were subsequently recruited into the cohort. Since age 7 years, the children attended annual clinical assessments during which they participated in various face-to-face interviews and physical tests. Detailed information about the ALSPAC cohort can be found on the study website (http://www.bristol.ac.uk/alspac), and the sample characteristics and methodology have been previously described (Boyd et al., 2013, Fraser et al., 2013). For information on all available ALSPAC data, a fully searchable data dictionary is also available (http://www.bris.ac.uk/alspac/researchers/data-access/data-dictionary).

The risk sets for the analyses presented here were comprised of 5074 and 5086 participants who provided enough blood during clinical assessments at age 9 years to measure serum IL-6 and CRP levels, respectively (Supplementary Fig. 1). 2731 participants with baseline IL-6 data had also attended the follow-up assessment for depressive symptoms at age 18 years; this analytic sample was used to assess associations between IL-6 and depressive symptoms. The analytic sample for CRP was similar (n = 2738). Ethical approval for the study was obtained from the ALSPAC Ethics and Law Committee and the Local Research Ethics Committees.

### 2.2 Measurement of inflammatory markers

Blood samples collected from non-fasting 9-year-old participants (mean age: 119.0 months; SD: 3.9 months) during clinical assessments were immediately centrifuged; serum samples were frozen at −80 °C in 1 mL aliquots. After a median storage period of 7.5 years, IL-6 and CRP levels were assayed in 2008. There was no evidence of freeze-thaw cycles during storage.

Serum high sensitivity CRP levels were measured using an automated particle-enhanced immunoturbidimetric assay (Roche, Welwyn Garden City, UK). The minimum detection limit was 0.03 mg/L. CRP values in the entire sample (n = 5086) ranged from 0.01 to 67.44 mg/L; mean (SD) = 0.80 (2.72) mg/L. Serum IL-6 levels were measured using an enzyme-linked immunosorbent assay (ELISA) (R&D Systems, UK). The minimum detection limit was 0.007 pg/mL. IL-6 values in the entire sample (n = 5074) ranged from 0.007 to 20.051 pg/mL; mean (SD) = 1.28 (1.59) pg/mL. All inter-assay and intra-assay coefficients of variation for IL-6 and CRP were <5%. No other inflammatory markers were measured from blood samples collected at age 9 years.

Serum IL-6 and CRP levels were analysed as categorical variables. The distributions of serum CRP values (n = 5086) and serum IL-6 values (n = 5074) were divided into three categories (bottom, middle and top thirds) based on their respective distribution tertiles (66th and 33rd percentiles for CRP were 0.37 and 0.14 mg/L, respectively; 66th and 33rd percentiles for IL-6 were 1.12 and 0.59 pg/mL, respectively). Statistical cut-offs were chosen due to the lack of accepted cut-off values for IL-6 and CRP in defining low-grade inflammation in children. We carried out additional analysis using IL-6 quartiles. Serum IL-6 and CRP levels were also analysed as standardised continuous variables (z-transformed values).

### 2.3 Psychiatric measures

#### 2.3.1 Assessment of depressive symptoms at age 18 years

In assessment clinics held over the course of a few months, depressive symptoms were assessed in 18-year-old participants (mean age: 213.6 months; SD: 5.1 months) using a self-administered computerized version of the Clinical Interview Schedule-Revised (CIS-R). CIS-R is a standardised and validated tool commonly used to assess depression and anxiety in community-based samples (Lewis et al., 1992). As primary outcomes, we used all available specific depressive symptom level data gathered by CIS-R as individual variables. A total of 19 depressive symptoms were measured: fatigue, sleep disturbances, concentration difficulties, irritability, depressed/low mood, suicidality, worry, phobia, anxiety, panic, psychomotor change, change in appetite/weight, diurnal variation in mood, anhedonia, hopelessness, low self-esteem, self-blame, decreased libido and physical symptoms. Because we were interested in clinically significant depressive symptoms, we re-coded all depressive symptom as binary variables based on a clinically meaningful cut-off. Please see Supplementary Methods for full information on the data re-coding. Supplementary Table 1 presents a complete breakdown of responses to each symptom category, as originally coded by CIS-R, and for the re-coded variables.

#### 2.3.2 Somatic/neurovegetative and psychological symptoms of depression

Somatic syndrome is recognised as a part of the diagnosis of depressive episode (F32) according to the ICD-10 diagnostic criteria for research (ICD-10: DCR-10) (WHO, 1992). We selected the following seven symptoms as somatic/neurovegetative based on this criteria: anhedonia, diurnal variation in mood, psychomotor change, change in appetite/weight, fatigue, sleep disturbances and decreased libido. The following seven symptoms were classified as psychological: depressed/low mood, concentration difficulties, irritability, hopelessness, self-blame, low self-esteem and suicidality. Each symptom was considered as a separate outcome variable. In addition, we carried out confirmatory factor analysis (CFA) to create two factor scores representing somatic/neurovegetative and psychological dimensions using the seven respective individual symptoms listed above.

### 2.4 Assessment of covariates

We included age, sex, father’s occupation, ethnicity, BMI and self-reported infections around blood collection as potential confounders. Because not every participant was exactly 18 years old at the time of outcome assessment, age was recorded at the time of outcome assessment in months and was used as a continuous variable. Sex was recorded at birth and treated as a binary variable. Father's occupation was recorded at birth according to the UK Office of National Statistics' socioeconomic classification system (Class I = professionals and higher managerial workers; II = intermediate occupations; IIIa = skilled non-manual occupations; IIIb = skilled manual occupations; IV = partly skilled occupations; V = unskilled occupations) (Statistics, 1980). This was re-coded as a binary variable with non-manual (I, II, and IIIa) and manual occupations (IIIb, IV, and V). Ethnicity was recorded at birth and originally coded as a categorical variable (White, Black Caribbean, Black African, Other Black, India, Pakistani, Bangladeshi, Chinese and Other). Ethnicity was re-coded as a binary variable (British White and Non-white) because the study sample was predominantly made up of British White participants (97.4%). BMI was assessed at the time of blood collection and recorded as a continuous variable. Self-reported infection status at the time or week before blood collection was recorded during the clinical assessment at age 9 years and used as a binary variable (i.e. infection present or absent).

### 2.5 Statistical analysis

#### 2.5.1 Comparison of baseline characteristics

Sociodemographic and other characteristics were compared among groups with low, medium and high IL-6 at age 9 years. One-way analysis of variance (ANOVA) was used for normally distributed continuous variables; Kruskal-Wallis test was used for non-normally distributed continuous variables; and Chi-squared test was used for categorical variables.

#### 2.5.2 Association between inflammatory markers and specific symptoms of depression

We used modified Poisson generalised linear regression with robust error variance to estimate the risk ratio (RR) and 95% confidence interval (CI) for each depressive symptom at age 18 years for participants in the top and middle, compared with the bottom, thirds of the serum IL-6 distribution at age 9 years. The same approach was taken for CRP. All regression models were adjusted for age, sex, father's occupation, ethnicity, BMI and self-reported infection. Adjusted RRs (95% CIs) were plotted on forest plots. *P-*values for unadjusted RRs for IL-6 were corrected for multiple testing using the Holm–Bonferroni method (Holm, 1979). This method is similar to the Bonferroni, yet less stringent. Some have argued that this method is better than Bonferroni for public health research (Aickin and Gensler, 1996). For a step-by-step guide on how this correction is calculated, along with an example, please see: http://nebc.nerc.ac.uk/courses/GeneSpring/GS_Mar2006/Multiple%20testing%20corrections.pdf.

#### 2.5.3 Association between inflammatory markers and symptom dimension scores

First, we carried out CFA to test whether a unidimensional or two-factor model provided better fit for the symptom data (7 somatic/neurovegetative and 7 psychological symptoms) using the full information maximum likelihood (FIML) estimator. FIML allows for direct comparison of both models. The results showed that two-factor model was marginally better than one-factor model (unidimensional: AIC = 42376, BIC = 42556; two-factor: AIC = 42351, BIC = 42538). We then added IL-6 into the model to test the association between IL-6 and two continuous latent variables/factors that represented somatic/neurovegetative and psychological dimensions. This was a structural equation model (SEM), which computed path coefficients, standard errors (SEs) and *P*-values for the association of IL-6 (standardised continuous variable) with these latent variables. The estimated coefficients are on the probit scale and represent changes in the symptom dimension standardised scores (Z-scores) per SD change in IL-6 levels. In addition, we used a T-test to examine whether coefficients for somatic/neurovegetative and psychological dimensions were significantly different from each other.

## 3 Results

### 3.1 Baseline characteristics of sample

Participants in the top third of IL-6 levels at baseline were more likely to be female, have higher BMI and have a self-reported infection around the time of blood collection at age 9 years. However, the distributions of age, British white ethnicity and father's non-manual occupations were similar across the thirds of IL-6 (Table 1).

**Table 1:** Baseline Characteristics of Participants by the Distribution of Serum IL-6 Values at Age 9 Years.

Characteristic	Distribution of Serum IL-6 Levels at Age 9 Years^a^			*P*-value^b^
	Bottom Third	Middle Third	Top Third	
Total no. of participants	1674	1675	1725	
Age (months) at outcome, mean (SD)^c^	212.9 (4.6)	213.2 (4.7)	213.3 (4.9)	0.11
Male sex, no. (%)	988 (59.0)	852 (50.9)	721 (41.8)	<0.001
BMI at age 9 years, median (IQR)^d^	16.4 (15.4–17.8)	17.0 (15.6–18.9)	17.7 (16.0–20.3)	<0.001
British white ethnicity, no. (%)^e^	1532 (98.4)	1506 (98.2)	1527 (97.8)	0.47
Father’s occupation, non-manual, no. (%)^f^	926 (63.6)	871 (61.3)	854 (60.0)	0.13
Self-reported infection at age 9 years, no. (%)^g^	83 (5.0)	149 (8.9)	248 (14.4)	<0.001

*Abbreviations:* BMI = Body Mass Index; IL-6 = Interleukin 6; IQR = Interquartile Range (range of values between the 25th and 75th percentile data values); No. = Number; SD = Standard Deviation

a Participants in the IL-6 risk set at age 9 years were divided into thirds based on the tertiles of the serum IL-6 distribution; the cut-offs for the top (66th percentile) and bottom (33rd percentile) tertiles were 1.12 and 0.59 pg/mL, respectively

b ANOVA was used to assess mean ages across the thirds of IL-6 at age 9 years; the Kruskal-Wallis test was used to assess median BMI at age 9 years across the thirds of IL-6 at age 9 years; the Chi-squared test for proportions was used to test equal proportions of categorical characteristics (sex, ethnicity, father’s occupation and infection status at age 9 years) between the thirds of IL-6 at age 9 years

c Data on age at age 18 years was available for only 2995 participants in the three categories; the sample sizes for the bottom, middle and top thirds were 990, 983 and 1022, respectively

d The median value and IQR for BMI at age 9 years was reported due to a lack of normality in the distribution of BMI values; data on BMI was available for only 5018 participants in the three categories; the sample sizes for the bottom, middle and top thirds were 1653, 1660, and 1705, respectively

e Data on ethnicity was available for only 4651 participants in the three categories; the denominators for the bottom, middle and top thirds were 1557, 1533, and 1561, respectively

f Data on father’s occupation was available for only 4300 participants in the three categories; the denominators for the bottom, middle and top thirds were 1455, 1422, and 1423, respectively

g Data on infection status at age 9 years was available for only 5065 participants in the three categories; the denominators for the bottom, middle and top thirds were 1672, 1672, and 1721, respectively

### 3.2 Prevalence of specific symptoms of depression at age 18 years

Data on 19 specific symptoms of depression were available for a total of 4568 participants. Fatigue (31.5%), hopelessness (31.1%) and change in appetite/weight (28.1%) were the most prevalent symptoms, while phobia (6.7%), decreased libido (4.5%) and panic (2.1%) were the least prevalent symptoms (Supplementary Table 1).

### 3.3 Association between inflammatory markers and specific symptoms of depression

**Fig. 1:**
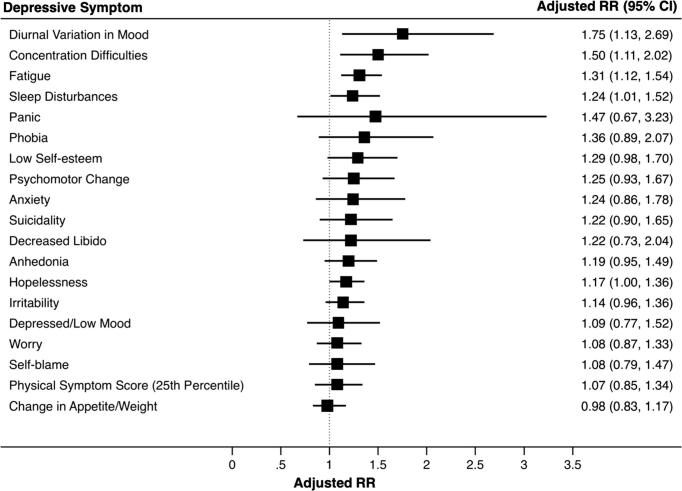
Adjusted risk ratios (RR) and 95% CIs for symptoms of depression at age 18 years for participants in the top, compared with bottom, third of serum IL-6 levels at age 9 years Footnote: The forest plot displays the adjusted RRs (95% CIs) for all 19 depressive symptoms at age 18 years when comparing participants in the top third with those in the bottom third of serum IL-6 levels at age 9 years. The RRs were adjusted for age at the time of outcome assessment, sex, ethnicity, father’s occupation, BMI at age 9 years and self-reported infection at age 9 years. Fatigue, sleep disturbances, concentration difficulties and diurnal variation in mood remained associated with IL-6 after adjusting for confounding.

**Fig. 2:**
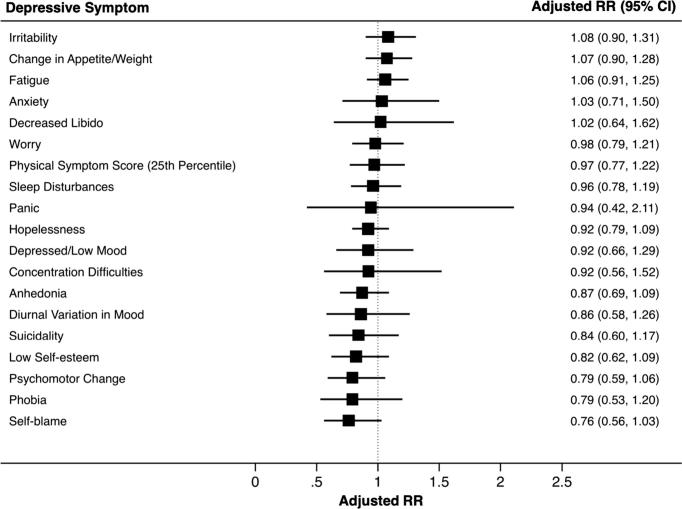
Adjusted risk ratios (RR) and 95% CIs for symptoms of depression at age 18 years for participants in the top, compared with bottom, third of serum CRP levels at age 9 years Footnote: The forest plot displays the adjusted RRs (95% CIs) for all 19 depressive symptoms at age 18 years when comparing participants in the top third with those in the bottom third of serum CRP levels at age 9 years. The RRs were adjusted for age at the time of outcome assessment, sex, ethnicity, father’s occupation, BMI at age 9 years and self-reported infection at age 9 years. CRP was not associated with depressive symptoms after adjusting for confounding.

IL-6 at age 9 years was associated with diurnal variation in mood, concentration difficulties, fatigue and sleep disturbances at age 18 years after adjusting for potential confounders (Fig. 1; Supplementary Table 2). The adjusted RRs (95% CI) for diurnal variation in mood, concentration difficulties, fatigue and sleep disturbances were 1.75 (1.13–2.69), 1.50 (1.11–2.02), 1.31 (1.12–1.54), and 1.24 (1.01–1.52), respectively, for participants in the top, compared with the bottom, third of IL-6 at age 9. CRP was associated with fatigue, change in appetite/weight, and irritability in the unadjusted analysis; however, these associations became attenuated after controlling for potential confounders (Fig. 2; Supplementary Table 3).

Sensitivity analyses using quartiles of IL-6 showed that IL-6 was associated with fatigue, diurnal variation in mood, concentration difficulties and hopelessness after controlling for potential confounders for participants in the top quartile of IL-6 distribution compared with those in the bottom quartile. There was also trend level association of IL-6 with anhedonia, psychomotor change and low self-esteem. Please see Supplementary Table 4.

### 3.4 Association between inflammatory markers and symptom dimension scores

**Fig. 3:**
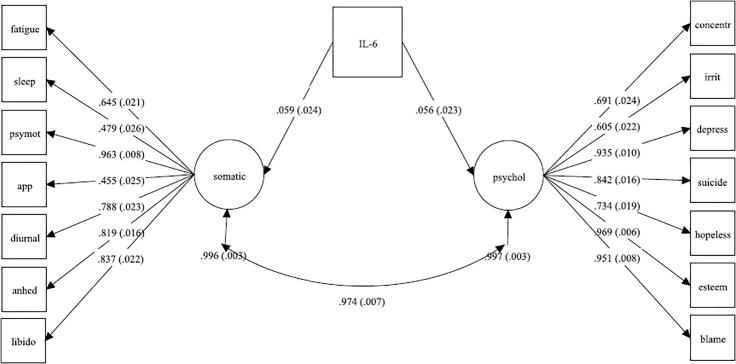
Structural Equation Model Testing the Association of IL-6 with Somatic/Neurovegetative and Psychological Symptom Latent Variables Footnote: Confirmatory factor analysis computed somatic and psychological factors, which were used as outcome variables in relation to IL-6 in SEM. The path co-efficient for the association between IL and 6 and the somatic factor was adjusted for the psychological factor, and *vice versa*.

SEM analysis testing the association between IL-6 and two latent variables representing depressive symptom dimensions showed that IL-6 was associated with both somatic/neurovegetative (co-efficient = 0.059, SE = 0.024, *P* = 0.013) and psychological dimensions (co-efficient = 0.056, SE = 0.023, *P* = 0.016); see Fig. 3. In other words, a SD change in IL-6 levels translated to a change in the Z-scores by 0.059 points for the somatic/neurovegetative dimension and 0.056 points for the psychological dimension. A *t*-test comparing the coefficients did not show a statistically significant difference (*P* = 0.379).

### 3.5 Effect of multiple testing

IL-6 was associated with 11 depressive symptoms before adjusting for potential confounders (Supplementary Table 2). Evidence for association remained for three symptoms (fatigue, sleep disturbances and hopelessness) after correcting observed *P-*values for multiple testing (Table 2).

**Table 2:** Corrected *P*-values for the Association between IL-6 and Depressive Symptoms using the Holm–Bonferroni Method.

Depressive Symptom	Original *P-*value^a^	Corrected *P-*value
Fatigue	3.6 × 10^−7	0.0000038
Sleep disturbances	0.001	0.018
Concentration difficulties	0.008	0.120
Irritability	0.016	0.208
Depressed/low mood	0.215	0.860
Suicidality	0.093	0.588
Worry	0.040	0.400
Phobia	0.020	0.240
Anxiety	0.118	0.590
Panic	0.988	0.988
Psychomotor change	0.027	0.297
Change in appetite/weight	0.293	0.879
Diurnal mood variation	0.052	0.468
Anhedonia	0.013	0.182
Hopelessness	0.001	0.018
Low self-esteem	0.004	0.064
Self-blame	0.084	0.588
Decreased libido	0.301	0.879
Physical symptoms	0.062	0.496

a The *P*-values correspond to unadjusted RRs presented in Supplementary Table 2: RR for each symptom at age 18 years for participants in the top, compared with bottom, third of the distribution of serum IL-6 levels at age 9.

### 3.6 Effect of missing data

To explore the potential impact of differential participant attrition over time, we compared participants in the IL-6 analytic sample (i.e. data present for both IL-6 and depressive symptom) with those in the missing sample (i.e. data present for IL-6 and missing depressive symptom). IL-6 levels, BMI, self-reported infection and British White ethnicity were similar between the analytic and missing samples (Online Supplementary Table 4). However, participants in the missing sample were more likely to be older, male and have a poorer socioeconomic status (fathers with manual occupations) compared to those in the analytic sample.

### 3.7 Effect of acute infection

We re-tested the associations between CRP and depressive symptoms after excluding 60 participants (1.2% of a total of 5086 participants) who had serum CRP levels >10 mg/L, an indicator of possible acute infection. The results remained virtually unchanged. CRP was not associated with any depressive symptom after adjusting for potential confounders (Online Supplementary Table 5).

## 4 Discussion

Using longitudinal data from a general population-representative birth cohort, we have examined associations between childhood IL-6 and CRP levels and specific symptoms of depression in early adulthood. Individual symptom level analysis indicated that IL-6 was associated with diurnal mood variation, concentration difficulties, fatigue and sleep disturbances after adjusting for potential confounders. Further analyses using latent variables showed that IL-6 was associated with both somatic/neurovegetative and psychological dimension scores. Adjusting for confounding had a more pronounced effect on the association between CRP and depressive symptoms; CRP was not associated with any depressive symptom after adjusting for confounders.

To our knowledge, this is one of the first general population-based longitudinal studies to investigate the association between inflammatory markers and specific depressive symptoms. Low-grade inflammation, as defined by elevated serum concentrations of IL-6 and CRP, is associated with the diagnosis of depression (Dowlati et al., 2010, Goldsmith et al., 2016, Haapakoski et al., 2015, Howren et al., 2009), depressive symptom severity (Jokela et al., 2016, Kohler-Forsberg et al., 2017, Moieni et al., 2015) and persistent depressive symptoms (Khandaker et al., 2017b, Zalli et al., 2016). Cross-sectional studies have reported an association between inflammatory markers and somatic symptoms of depression such as fatigue, sleep disturbances and change in appetite and activity levels (Case and Stewart, 2014, Jokela et al., 2016, Kohler-Forsberg et al., 2017). We have replicated some of these findings using a longitudinal design where inflammatory markers were measured years before depressive symptoms developed. These findings are consistent with existing literature, which suggests that inflammation may be a cause for depressive symptoms, rather than simply be a consequence of the illness (Gimeno et al., 2009, Khandaker et al., 2014, Khandaker et al., 2017a).

In our analysis, inflammatory cytokine IL-6 was associated with so-called somatic/neurovegetative symptoms such as fatigue, sleep disturbances and diurnal variation in mood. This is consistent with findings from the NESDA cohort, which reported that inflammatory markers were specifically associated with somatic symptoms of depression such as fatigue, weight gain and sleep disturbances (Duivis et al., 2013). Similarly, immuno-activation in animals and healthy volunteers leads to fatigue, psychomotor retardation and anhedonia, which are typical somatic symptoms according to ICD-10 diagnostic criteria for depressive episode (Brydon et al., 2008, Capuron et al., 2012, Eisenberger et al., 2010, Harrison et al., 2009, Harrison et al., 2015). Features of sickness behavior (e.g., fatigue) develop rapidly in the majority of interferon-treated cancer patients who develop depression, while psychological symptoms (e.g., low mood) develop slowly and relatively less frequently (Capuron et al., 2009, Capuron et al., 2002). Inflammation-induced somatic/neurovegetative symptoms could be important mediators of the relationship between inflammation and depression. Experimental studies including clinical trials in humans are required to test this hypothesis.

One possibility is that somatic/neurovegetative symptoms affect mood by altering reward perception. Although the association between IL-6 and anhedonia was no longer statistically significant after adjusting for confounding within our dataset, previous experimental studies have reported an association between inflammation and anhedonia. In non-human primates chronic low-dose infusion of interferon-alpha leads to reduction in striatal dopamine release and anhedonia-like behavior (Felger et al., 2013). In patients with depression, plasma CRP concentration is associated with left basal ganglia glutamate levels, which, in turn, is associated with psychomotor slowing and anhedonia (Felger et al., 2016, Haroon et al., 2016). In healthy volunteers, experimental immuno-activation alters activation of reward-related brain regions (Brydon et al., 2008, Capuron et al., 2012, Eisenberger et al., 2010, Harrison et al., 2009, Harrison et al., 2015), including reduction in the ventral striatum responses to hedonic reward (Capuron et al., 2012, Eisenberger et al., 2010). In the future, RCTs and animal experimental studies could test whether altered reward perception mediate the relationship between somatic/neurovegetative symptoms and cognitive/psychological symptoms of depression following immuno-modulation.

Association of inflammation with specific symptoms indicate that these symptoms could be important treatment targets and markers of treatment response in clinical trials of anti-inflammatory drugs for depression. Selecting patients based on inflammation levels may be a fruitful strategy for clinical trials of anti-inflammatory drugs (Kappelmann et al., 2018, Raison et al., 2013). Similarly, focusing on specific symptoms or symptom dimensions as outcomes may also be useful. Somatic/neurovegetative symptoms such as fatigue and sleep disturbances have considerable negative impact on daily functioning and quality of life in patients with physical illness (Janardhan and Bakshi, 2002). There is little work on the impact of somatic/neurovegetative symptoms on quality of life in patients with depression. Studies exploring the burden and impact of these symptoms in patients with depression are required.

We have defined certain depressive symptoms as ‘somatic/neurovegetative’ based on the ICD-10: DCR-10 criteria for depressive episode, somatic syndrome (see Methods) (WHO, 1992). This is also informed by the experimental animal literature, where certain inflammation-induced symptoms such as anorexia, decreased motor activity, loss of interest and sleep disturbances are often described as sickness behavior or neurovegetative symptoms (Dantzer et al., 2008). The ICD-10 somatic symptoms of depression can be broadly seen as the human analogue of animal neurovegetative symptoms because these symptoms overlap, albeit not completely. However, we acknowledge that there are inconsistencies in the use of the term ‘somatic’ in the literature. It has also been used to refer to bodily distress, such as palpitations, tightness of the chest, pain, etc. (Kapfhammer, 2006). This is not the same as the somatic syndrome currently defined by ICD-10: DCR:10 criteria for depression. Our use of the term is conceptually aligned with the ICD-10 definition of somatic syndrome in humans and inflammation-induced neurovegetative symptoms in animal studies, rather than bodily distress reflecting worry.

Strengths of this study include a relatively large general population-based sample, longitudinal design and assessment of an array of depressive symptoms, 19 in total. However, there are some key limitations. Firstly, CRP was not associated with any depressive symptom which was surprising since IL-6 is a key inducer of CRP (Janeway et al., 2001). The use of non-fasting blood samples may have increased measurement error and is likely to have biased the findings toward the null. We adjusted our results for a number of potential confounders, but residual confounding could still be an alternative explanation for the observed associations between IL-6 and somatic symptoms. However, our work using Mendelian randomization analysis suggest that the association between IL-6/CRP and depression is likely to be causal (Khandaker et al., 2018a, Khandaker et al., 2018b).

Serum IL-6 and CRP levels in our sample were generally low (Section 2.2), which could be due to the young age of the participants. The reported mean serum CRP levels in adults (mean age: 57 years) within a large community-based Danish study were higher in comparison with those in the relatively young ALSPAC sample aged 9 years. In the Danish sample, CRP levels further increased after a 10-year follow-up period (Wium-Andersen et al., 2013).

Another significant limitation is the attrition of study participants between the time points of blood collection and depressive symptom assessment. Missingness was associated with older age, male sex and poorer SES. Older age and lower SES are both associated with depression. An over-representation of individuals within these groups within the missing sample could introduce a bias towards the null. Therefore, we are likely to have underestimated the true association between IL and 6 and specific depressive symptoms.

In summary, our findings indicate that childhood IL-6 levels are associated with specific symptoms of depression in early adulthood. Associations with so-called somatic/neurovegetative symptoms such as fatigue, sleep disturbances and diurnal mood variation indicate that these symptoms could be useful treatment targets and markers of treatment response in clinical trials of anti-inflammatory drugs for depression.

## Declaration of interest

None of the authors has any conflicts of interest to declare.

## Funding

The UK Medical Research Council and Wellcome (Grant ref: 102215/2/13/2) and the University of Bristol provide core support for ALSPAC. This publication is the work of the authors, and Dr Khandaker and Mr Chu will serve as guarantors for the contents of this paper. Dr Khandaker is supported by an Intermediate Clinical Fellowship from the Wellcome Trust (201486/Z/16/Z), a Clinical Lecturer Starter Grant from the Academy of Medical Sciences, UK (grant no. 80354), and a Data Science Award from the MQ: Transforming Mental Health (grant no. MQDS17/40). Prof Jones acknowledges grant support from the Wellcome Trust (095844/Z/11/Z & 088869/Z/09/Z) and NIHR (RP-PG-0606-1335). Prof Zammit acknowledges funding support from the National Institute for Health Research Biomedical Research Centre at the University Hospitals Bristol National Health Service Foundation Trust and the University of Bristol. The funding bodies had no role in design and conduct of the study; collection, management, analysis, and interpretation of the data; preparation, review, or approval of the manuscript; and decision to submit the manuscript for publication.

## References

[b0005] Aickin M., Gensler H. (1996). Adjusting for multiple testing when reporting research results: the Bonferroni vs Holm methods. Am. J. Public Health.

[b0010] Boyd A., Golding J., Macleod J., Lawlor D.A., Fraser A., Henderson J., Molloy L., Ness A., Ring S., Davey Smith G. (2013). Cohort Profile: the 'Children of the 90s'–the index offspring of the Avon Longitudinal Study of Parents and Children. Int. J. Epidemiol..

[b0015] Brydon L., Harrison N.A., Walker C., Steptoe A., Critchley H.D. (2008). Peripheral inflammation is associated with altered substantia nigra activity and psychomotor slowing in humans. Biol. Psychiatry.

[b0020] Capuron L., Gumnick J.F., Musselman D.L., Lawson D.H., Reemsnyder A., Nemeroff C.B., Miller A.H. (2002). Neurobehavioral effects of interferon-alpha in cancer patients: phenomenology and paroxetine responsiveness of symptom dimensions. Neuropsychopharmacology.

[b0025] Capuron L., Fornwalt F.B., Knight B.T., Harvey P.D., Ninan P.T., Miller A.H. (2009). Does cytokine-induced depression differ from idiopathic major depression in medically healthy individuals?. J. Affect. Disord..

[b0030] Capuron L., Pagnoni G., Drake D.F., Woolwine B.J., Spivey J.R., Crowe R.J., Votaw J.R., Goodman M.M., Miller A.H. (2012). Dopaminergic mechanisms of reduced basal ganglia responses to hedonic reward during interferon alfa administration. Arch. Gen. Psychiatry.

[b0035] Case S.M., Stewart J.C. (2014). Race/ethnicity moderates the relationship between depressive symptom severity and C-reactive protein: 2005–2010 NHANES data. Brain Behav. Immun..

[b0040] Dantzer R., O'Connor J.C., Freund G.G., Johnson R.W., Kelley K.W. (2008). From inflammation to sickness and depression: when the immune system subjugates the brain. Nat. Rev. Neurosci..

[b0045] Dowlati Y., Herrmann N., Swardfager W., Liu H., Sham L., Reim E.K., Lanctot K.L. (2010). A meta-analysis of cytokines in major depression. Biol. Psychiatry.

[b0050] Duivis H.E., Vogelzangs N., Kupper N., de Jonge P., Penninx B.W. (2013). Differential association of somatic and cognitive symptoms of depression and anxiety with inflammation: findings from the Netherlands Study of Depression and Anxiety (NESDA). Psychoneuroendocrinology.

[b0055] Eisenberger N.I., Berkman E.T., Inagaki T.K., Rameson L.T., Mashal N.M., Irwin M.R. (2010). Inflammation-induced anhedonia: endotoxin reduces ventral striatum responses to reward. Biol. Psychiatry.

[b0060] Felger J.C., Mun J., Kimmel H.L., Nye J.A., Drake D.F., Hernandez C.R., Freeman A.A., Rye D.B., Goodman M.M., Howell L.L., Miller A.H. (2013). Chronic interferon-alpha decreases dopamine 2 receptor binding and striatal dopamine release in association with anhedonia-like behavior in nonhuman primates. Neuropsychopharmacology.

[b0065] Felger J.C., Li Z., Haroon E., Woolwine B.J., Jung M.Y., Hu X., Miller A.H. (2016). Inflammation is associated with decreased functional connectivity within corticostriatal reward circuitry in depression. Mol. Psychiatry.

[b0070] Fraser A., Macdonald-Wallis C., Tilling K., Boyd A., Golding J., Davey Smith G., Henderson J., Macleod J., Molloy L., Ness A., Ring S., Nelson S.M., Lawlor D.A. (2013). Cohort Profile: the Avon Longitudinal Study of parents and children: ALSPAC mothers cohort. Int. J. Epidemiol..

[b0075] Gimeno D., Kivimaki M., Brunner E.J., Elovainio M., De Vogli R., Steptoe A., Kumari M., Lowe G.D., Rumley A., Marmot M.G., Ferrie J.E. (2009). Associations of C-reactive protein and interleukin-6 with cognitive symptoms of depression: 12-year follow-up of the Whitehall II study. Psychol. Med..

[b0080] Goldsmith D.R., Rapaport M.H., Miller B.J. (2016). A meta-analysis of blood cytokine network alterations in psychiatric patients: comparisons between schizophrenia, bipolar disorder and depression. Mol. Psychiatry.

[b0085] Haapakoski R., Mathieu J., Ebmeier K.P., Alenius H., Kivimaki M. (2015). Cumulative meta-analysis of interleukins 6 and 1beta, tumour necrosis factor alpha and C-reactive protein in patients with major depressive disorder. Brain Behav. Immun..

[b0090] Haroon E., Fleischer C.C., Felger J.C., Chen X., Woolwine B.J., Patel T., Hu X.P., Miller A.H. (2016). Conceptual convergence: increased inflammation is associated with increased basal ganglia glutamate in patients with major depression. Mol. Psychiatry.

[b0095] Harrison N.A., Brydon L., Walker C., Gray M.A., Steptoe A., Critchley H.D. (2009). Inflammation causes mood changes through alterations in subgenual cingulate activity and mesolimbic connectivity. Biol. Psychiatry.

[b0100] Harrison N.A., Cercignani M., Voon V., Critchley H.D. (2015). Effects of inflammation on hippocampus and substantia nigra responses to novelty in healthy human participants. Neuropsychopharmacology.

[b0105] Holm S. (1979). A simple sequentially rejective multiple test procedure. Scand J Statist.

[b0110] Howren M.B., Lamkin D.M., Suls J. (2009). Associations of depression with C-reactive protein, IL-1, and IL-6: a meta-analysis. Psychosom. Med..

[b0115] Janardhan V., Bakshi R. (2002). Quality of life in patients with multiple sclerosis: the impact of fatigue and depression. J. Neurol. Sci..

[b0120] Janeway C.A., Travers P., Walport M., Shlomchik M.J. (2001). Immunobiology: The Immune System in Health and Disease.

[b0125] Jokela M., Virtanen M., Batty G.D., Kivimaki M. (2016). Inflammation and specific symptoms of depression. JAMA Psychiatry.

[b0130] Kapfhammer H.P. (2006). Somatic symptoms in depression. Dialogues Clin. Neurosci..

[b0135] Kappelmann N., Lewis G., Dantzer R., Jones P.B., Khandaker G.M. (2018). Antidepressant activity of anti-cytokine treatment: a systematic review and meta-analysis of clinical trials of chronic inflammatory conditions. Mol. Psychiatry.

[b0140] Khandaker, G.M., Zuber, V., Rees, J.M.B., Carvalho, L., Mason, A.M., Foley, C.N., Gkatzionis, A., Jones, P.B., Burgess, S., 2018b. Shared mechanism between depression and coronary heart disease: findings from Mendelian randomization analysis of a large UK population-based cohort (under review).

[b0145] Khandaker G.M., Pearson R.M., Zammit S., Lewis G., Jones P.B. (2014). Association of serum interleukin 6 and C-reactive protein in childhood with depression and psychosis in young adult life: a population-based longitudinal study. JAMA Psychiatry.

[b0150] Khandaker G.M., Stochl J., Zammit S., Goodyer I., Lewis G., Jones P.B. (2017). Childhood inflammatory markers and intelligence as predictors of subsequent persistent depressive symptoms: a longitudinal cohort study. Psychol. Med..

[b0155] Khandaker G.M., Stochl J., Zammit S., Goodyer I.M., Lewis G., Jones P.B. (2017). Childhood inflammatory markers and intelligence as predictors of subsequent persistent depressive symptoms: a longitudinal cohort study. Psychol. Med..

[b0160] Khandaker G.M., Zammit S., Burgess S., Lewis G., Jones P.B. (2018). Association between a functional interleukin 6 receptor genetic variant and risk of depression and psychosis in a population-based birth cohort. Brain Behav. Immun..

[b0165] Kohler-Forsberg O., Buttenschon H.N., Tansey K.E., Maier W., Hauser J., Dernovsek M.Z., Henigsberg N., Souery D., Farmer A., Rietschel M., McGuffin P., Aitchison K.J., Uher R., Mors O. (2017). Association between C-reactive protein (CRP) with depression symptom severity and specific depressive symptoms in major depression. Brain Behav. Immun..

[b0170] Lewis G., Pelosi A.J., Araya R., Dunn G. (1992). Measuring psychiatric disorder in the community: a standardized assessment for use by lay interviewers. Psychol. Med..

[b0175] Maes M., Bosmans E., De Jongh R., Kenis G., Vandoolaeghe E., Neels H. (1997). Increased serum IL-6 and IL-1 receptor antagonist concentrations in major depression and treatment resistant depression. Cytokine.

[b0180] Milaneschi Y., Lamers F., Peyrot W.J., Baune B.T., Breen G., Dehghan A., Forstner A.J., Grabe H.J., Homuth G., Kan C., Lewis C., Mullins N., Nauck M., Pistis G., Preisig M., Rivera M., Rietschel M., Streit F., Strohmaier J., Teumer A., Van der Auwera S., Wray N.R., Boomsma D.I., Penninx B., Group C.I.W., the Major Depressive Disorder Working Group of the Psychiatric Genomics, C. (2017). Genetic association of major depression with atypical features and obesity-related immunometabolic dysregulations. JAMA Psychiatry.

[b0185] Miller A.H., Maletic V., Raison C.L. (2009). Inflammation and its discontents: the role of cytokines in the pathophysiology of major depression. Biol. Psychiatry.

[b0190] Moieni M., Irwin M.R., Jevtic I., Olmstead R., Breen E.C., Eisenberger N.I. (2015). Sex differences in depressive and socioemotional responses to an inflammatory challenge: implications for sex differences in depression. Neuropsychopharmacology.

[b0195] O'Brien S.M., Scully P., Fitzgerald P., Scott L.V., Dinan T.G. (2007). Plasma cytokine profiles in depressed patients who fail to respond to selective serotonin reuptake inhibitor therapy. J. Psychiatr. Res..

[b0200] Raison C.L., Capuron L., Miller A.H. (2006). Cytokines sing the blues: inflammation and the pathogenesis of depression. Trends Immunol.

[b0205] Raison C.L., Rutherford R.E., Woolwine B.J., Shuo C., Schettler P., Drake D.F., Haroon E., Miller A.H. (2013). A randomized controlled trial of the tumor necrosis factor antagonist infliximab for treatment-resistant depression: the role of baseline inflammatory biomarkers. JAMA Psychiatry.

[b0210] Statistics, O.o.N., 1980. Classification of occupations and coding index. Office of Population Censuses and Surveys. Her Majesty's Stationery Office, London, pp. x. http://www.ons.gov.uk/ons/guide-method/classifications/archived-standard-classifications/soc-and-sec-archive/index.html.

[b0215] WHO (1992). The ICD-10 Classification of Mental and Behavioural Disorder: Clinical Descriptions and Diagnostic Guidelines.

[b0220] Wium-Andersen M.K., Orsted D.D., Nielsen S.F., Nordestgaard B.G. (2013). Elevated C-reactive protein levels, psychological distress, and depression in 73, 131 individuals. JAMA Psychiatry.

[b0225] Zalli A., Jovanova O., Hoogendijk W.J., Tiemeier H., Carvalho L.A. (2016). Low-grade inflammation predicts persistence of depressive symptoms. Psychopharmacology.

