# Supplementary material for: Longitudinal association between inflammatory markers and specific symptoms of depression in a prospective birth cohort
Source: Brain Behav Immun. 2019 Feb;76:74–81. doi: 10.1016/j.bbi.2018.11.007 (PMC6363967; doi:10.1016/j.bbi.2018.11.007)
Supplement: Supplementary Data 1 [file mmc1.docx]

**Online Supplementary Material**

**Chu *et al.* Longitudinal Association between Inflammatory Markers and Specific Symptoms of Depression in a Prospective Birth Cohort**

**Supplementary Methods:**

**Re-coding of CIS-R Depressive Symptom Data at Age 18 Years**

Hopelessness, low self-esteem and decreased libido were originally coded as binary variables – we used them in their original form. As shown in Supplementary Table 1 below, the following symptoms were coded originally on a five-point scale (0, 1, 2, 3 and 4) to represent the different symptom severities (0 = symptoms absent; 4 = most severe symptoms): fatigue; sleep disturbances; concentration difficulties; irritability; depressed/low mood; suicidality; worry; phobia; anxiety; and panic. To define clinically meaningful depressive symptoms, we re-coded these symptoms as binary variables by using a symptom score cut-off ≥2 after consulting with Professor Glyn Lewis, the co-developer of CIS-R. Creating two groups simply based on the presence or absence of depressive symptoms (score 0 vs. scores 1 - 4) could be misleading, because some of the more prevalent symptoms were reported by a relatively large proportion of the sample. For example, > 40% of the sample scored 1 or more for fatigue; > 35% of the sample scored 1 or more for sleep disturbances; about 35% of sample scored 1 or more for suicidality.

On the other hand, psychomotor change, change in appetite/weight, diurnal variation in mood and anhedonia were all originally coded with distinct categories and were not based on a five-point ordinal scale. We re-coded these variables as binary as follows. For psychomotor change, participants reporting psychomotor agitation or retardation were grouped together to represent psychomotor change. For change in appetite/weight, participants who reported loss in appetite, weight loss or weight gain were grouped as having experienced a change in appetite/weight. For diurnal variation in mood, participants who reported worst depressive symptoms in the morning or in the evening were grouped as having exhibited diurnal variation in mood. For anhedonia, participants who exhibited less enjoyment than usual or who did not enjoy anything in the past month were grouped as having experienced anhedonia. For self-blame, participants who reported to have experienced self-blame sometimes or often were grouped as having experienced self-blame.

Lastly, a total physical symptom score, the sum of the individual physical symptom scores for nausea, indigestion/stomach ache, joint pains, muscle pain, headaches, chest pains, throat pains, pain in the neck/armpit regions and dizziness/poor balance, was also available. We re-coded the total score as a binary variable by defining individuals with total scores ≥7 as exhibiting a high physical symptoms score; this threshold represented the top 25th percentiles of the total physical symptom scores.

**Supplementary Results:**

Supplementary Figure 1: Flow Diagram of Study Participants

Supplementary Table 1: Frequency of Specific Depressive Symptoms at Age 18 Years

Supplementary Table 2: Risk Ratios (95% CIs) for Specific Depressive Symptoms at Age 18 Years for Serum IL-6 Levels at Age 9 Years

Supplementary Table 3: Risk Ratios (95% CIs) for Specific Symptoms of Depression at Age 18 Years for Serum CRP Levels at Age 9 Years

Supplementary Table 4: Risk Ratios (95% CIs) for Specific Depressive Symptoms at Age 18 Years for Serum IL-6 Levels at Age 9 Years (Using IL-6 Quartiles)

Supplementary Table 5: Comparison of Baseline Characteristics Between the IL-6 Analytic (n = 2731) and Missing Data (n = 2343) Samples at Age 9 Years

Supplementary Table 6: Risk Ratios (95% CIs) for Specific Symptoms of Depression at Age 18 Years for Serum CRP Levels at Age 9 Years After Excluding Participants with CRP >10 mg/L (n = 60)


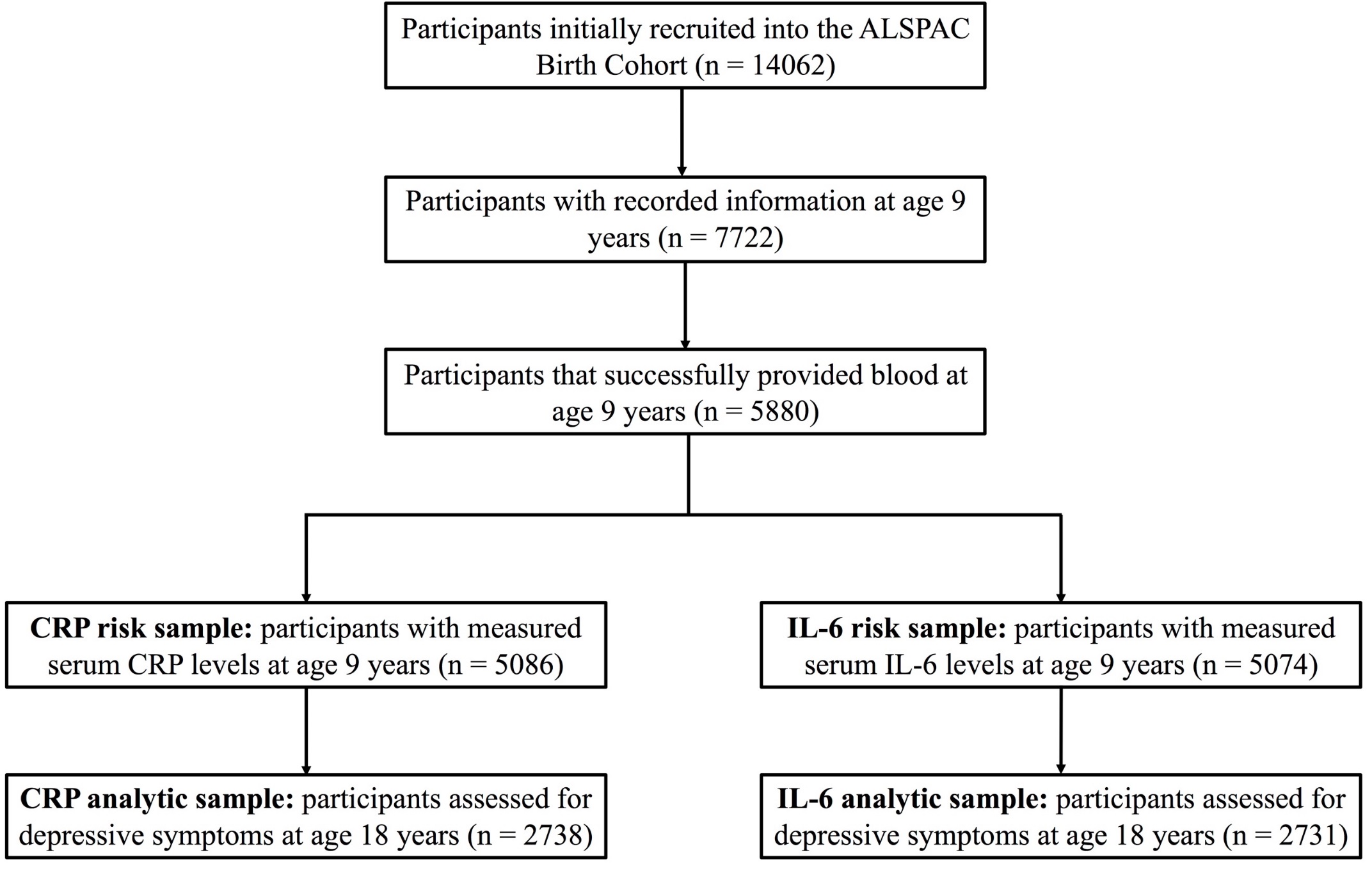


**Supplementary Figure 1: Flow Diagram of Study Participants**

Footnote: The total number of participants recruited into the ALSPAC birth cohort is based on those recruited during the initial (primary) phase between 1991 and 1992. The majority of participants that successfully provided blood at age 9 years had both measurable serum CRP and IL-6 levels (i.e. there is overlap between the CRP and IL-6 risk/analytic samples). The risk sample represents the total number of participants with measured serum inflammatory marker levels. The analytic sample represents the total number of participants with information on both their measured serum inflammatory marker levels at baseline and depressive symptoms at follow-up.

**Supplementary Table 1: Frequency of Specific Depressive Symptoms at Age 18 Years**

| **Depressive Symptom** | **Total No. of Participants** | **Prevalence of Symptom Score** $\boldsymbol{\geq}$**2, No. (%)^a^** | **Symptom Score/Code, No. (%)** | | | | |
| --- | --- | --- | --- | --- | --- | --- | --- |
|  |  |  | **0** | **1** | **2** | **3** | **4** |
| Fatigue | 4568 | 1439 (31.5) | 2636 (57.7) | 493 (10.8) | 468 (10.3) | 475 (10.4) | 496 (10.9) |
| Sleep disturbances | 4568 | 1106 (24.2) | 2881 (63.1) | 581 (12.7) | 735 (16.1) | 283 (6.2) | 88 (1.9) |
| Concentration difficulties | 4568 | 499 (10.9) | 3568 (78.1) | 501 (11.0) | 296 (6.5) | 141 (3.1) | 62 (1.4) |
| Irritability | 4568 | 1278 (28.0) | 2521 (55.2) | 769 (16.8) | 740 (16.2) | 345 (7.6) | 193 (4.2) |
| Depressed/low mood | 4568 | 463 (10.1) | 3589 (78.6) | 516 (11.3) | 245 (5.4) | 135 (3.0) | 83 (1.8) |
| Suicidality | 4568 | 501 (11.0) | 3002 (65.7) | 1065 (23.3) | 393 (8.6) | 108 (2.4) | - |
| Worry | 4568 | 962 (21.1) | 2730 (59.8) | 876 (19.2) | 436 (9.5) | 298 (6.5) | 228 (5.0) |
| Phobia | 4568 | 308 (6.7) | 3875 (84.8) | 385 (8.4) | 234 (5.1) | 53 (1.2) | 21 (0.5) |
| Anxiety | 4568 | 355 (7.8) | 3833 (83.9) | 380 (8.3) | 185 (4.1) | 106 (2.3) | 64 (1.4) |
| Panic | 4568 | 98 (2.1) | 4426 (96.9) | 44 (1.0) | 51 (1.1) | 31 (0.7) | 16 (0.4) |
| Psychomotor change | 4581 | 581 (12.7)^b^ | 4000 (87.3) | 406 (8.9) | 175 (3.8) | - | - |
| Change in appetite/weight | 4568 | 1282 (28.1)^c^ | 3286 (72.0) | 564 (12.4) | 557 (12.2) | 68 (1.5) | 93 (2.0) |
| Diurnal variation in mood | 4581 | 311 (6.8)^d^ | 4270 (93.2) | 61 (1.3) | 250 (5.5) | - | - |
| Anhedonia | 4568 | 860 (18.8)^e^ | 3708 (81.2) | 795 (17.4) | 65 (1.4) | - | - |
| Hopelessness | 4568 | 1419 (31.1)^f^ | 3149 (68.9) | 1419 (31.1) | - | - | - |
| Low self-esteem | 4581 | 619 (13.5)^g^ | 3962 (86.5) | 619 (13.5) | - | - | - |
| Self-blame | 4581 | 547 (12.0)^h^ | 3825 (83.5) | 209 (4.6) | 358 (7.8) | 189 (4.1) | - |
| Decreased libido | 4581 | 208 (4.5)^i^ | 4373 (95.5) | 208 (4.5) | - | - | - |
| Physical symptom score (top 25^th^ percentile) | 4568 | 839 (18.4)^j^ | 3729 (81.6) | 839 (18.4) | - | - | - |

^a^ Number (%) of individuals expressing depressive symptoms were derived by using a binary cut-off for all depressive symptoms; participants with symptom severity scores $\geq$2 were classified as exhibiting the depressive symptom while participants with symptom scores 0 or 1 were classified as not exhibiting the depressive symptom, unless otherwise specified (see below)

^b^ Derived from individuals who reported psychomotor agitation (symptom score 1) and retardation (symptom score 2)

^c^ Derived from individuals who reported a loss in appetite (symptom score 1), losing/gaining less than 7 pounds (symptom score 2), losing more than 7 pounds (symptom score 3) and gaining more than 7 pounds (symptom score 4)

^d^ Derived from individuals who reported worse depressive symptoms during the morning (symptom score 1) and evening (symptom score 2)

^e^ Derived from individuals who reported less enjoyment than usual (symptom score 1) and those who did not enjoy anything (symptom score 2)

^f^ Derived from individuals who reported hopelessness (symptom score 1)

^g^ Derived from individuals who experienced low self-esteem (symptom score 1)

^h^ Derived from individuals who reported self-blame sometimes (symptom score 2) and often (symptom score 3); individuals who experienced self-blame when deserved (symptom score 1) were classified as not exhibiting symptoms of self-blame (symptom score 0)

^i^ Derived from individuals who experienced decreased libido (symptom score 1)

^j^ Derived from individuals who had a physical symptom severity score between 7 to 24 out of 24 (symptom score 1); recorded physical symptoms included nausea, indigestion/stomach ache, joint pains, muscle pain, headaches, chest pains, throat pains, pain in the neck/armpit regions and dizziness/poor balance

Abbreviations: No.=Number

**Supplementary Table 2: Risk Ratios (95% CIs) for Specific Depressive Symptoms at Age 18 Years for Serum IL-6 Levels at Age 9 Years**

| **Depressive Symptom** | **IL-6 Group^a^** | **Sample** | **No. (%) with Symptom** | **Risk Ratio (95% CI) for Depressive Symptom** | | |
| --- | --- | --- | --- | --- | --- | --- |
|  |  |  |  | **Unadjusted** | **Adjusted for Age at Outcome, Sex, Father’s Occupation and Ethnicity** | **Further Adjusted for BMI and Self-reported Infections** |
| Fatigue | Bottom third | 905 | 234 (25.9) | 1 [Reference] | 1 [Reference] | 1 [Reference] |
|  | Middle third | 906 | 274 (30.2) | 1.17 (1.01 - 1.36) | 1.13 (0.96 - 1.32) | 1.12 (0.96 - 1.32) |
|  | Top third | 914 | 339 (37.1) | 1.43 (1.25 - 1.65) | 1.32 (1.13 - 1.53) | 1.31 (1.12 - 1.54) |
| Sleep disturbances | Bottom third | 905 | 167 (18.5) | 1 [Reference] | 1 [Reference] | 1 [Reference] |
|  | Middle third | 906 | 219 (24.2) | 1.31 (1.10 - 1.57) | 1.34 (1.11 - 1.63) | 1.34 (1.10 - 1.63) |
|  | Top third | 914 | 228 (24.9) | 1.35 (1.13 - 1.61) | 1.28 (1.05 - 1.56) | 1.24 (1.01 - 1.52) |
| Concentration difficulties | Bottom third | 905 | 82 (9.1) | 1 [Reference] | 1 [Reference] | 1 [Reference] |
|  | Middle third | 906 | 99 (10.9) | 1.21 (0.91 - 1.59) | 1.15 (0.85 - 1.56) | 1.15 (0.85 - 1.56) |
|  | Top third | 914 | 119 (13.0) | 1.44 (1.10 - 1.87) | 1.45 (1.09 - 1.94) | 1.50 (1.11 - 2.02) |
| Irritability | Bottom third | 905 | 221 (244) | 1 [Reference] | 1 [Reference] | 1 [Reference] |
|  | Middle third | 906 | 239 (26.4) | 1.08 (0.92 - 1.27) | 1.05 (0.89 - 1.24) | 1.05 (0.88 - 1.24) |
|  | Top third | 914 | 269 (29.4) | 1.21 (1.03 - 1.40) | 1.16 (0.98 - 1.37) | 1.14 (0.96 - 1.36) |
| Depressed/low mood | Bottom third | 905 | 74 (8.2) | 1 [Reference] | 1 [Reference] | 1 [Reference] |
|  | Middle third | 906 | 97 (10.7) | 1.31 (0.98 - 1.75) | 1.21 (0.88 - 1.66) | 1.21 (0.88 - 1.67) |
|  | Top third | 914 | 90 (9.8) | 1.20 (0.90 - 1.62) | 1.07 (0.77 - 1.48) | 1.09 (0.77 - 1.52) |
| Suicidality | Bottom third | 905 | 84 (9.3) | 1 [Reference] | 1 [Reference] | 1 [Reference] |
|  | Middle third | 906 | 87 (9.6) | 1.03 (0.78 - 1.38) | 0.90 (0.66 - 1.25) | 0.90 (0.65 - 1.24) |
|  | Top third | 914 | 107 (11.7) | 1.26 (0.96 - 1.65) | 1.21 (0.90 - 1.62) | 1.22 (0.90 - 1.65) |
| Worry | Bottom third | 905 | 164 (18.1) | 1 [Reference] | 1 [Reference] | 1 [Reference] |
|  | Middle third | 906 | 178 (19.6) | 1.08 (0.90 - 1.31) | 1.01 (0.83 - 1.24) | 1.01 (0.82 - 1.24) |
|  | Top third | 914 | 201 (22.0) | 1.21 (1.01 - 1.46) | 1.12 (0.91 - 1.36) | 1.08 (0.87 - 1.33) |
| Phobia | Bottom third | 905 | 41 (4.5) | 1 [Reference] | 1 [Reference] | 1 [Reference] |
|  | Middle third | 906 | 51 (5.6) | 1.24 (0.83 - 1.86) | 1.14 (0.74 - 1.75) | 1.15 (0.75 - 1.77) |
|  | Top third | 914 | 65 (7.1) | 1.57 (1.07 - 2.30) | 1.32 (0.88 - 1.99) | 1.36 (0.89 - 2.07) |
| Anxiety | Bottom third | 905 | 57 (6.3) | 1 [Reference] | 1 [Reference] | 1 [Reference] |
|  | Middle third | 906 | 63 (7.0) | 1.10 (0.78 - 1.56) | 1.03 (0.71 - 1.50) | 1.04 (0.71 - 1.50) |
|  | Top third | 914 | 75 (8.2) | 1.30 (0.93 - 1.82) | 1.22 (0.86 - 1.74) | 1.24 (0.86 - 1.78) |
| Panic | Bottom third | 909 | 15 (1.7) | 1 [Reference] | 1 [Reference] | 1 [Reference] |
|  | Middle third | 908 | 16 (1.8) | 1.07 (0.53 - 2.15) | 1.10 (0.48 - 2.54) | 1.19 (0.51 - 2.76) |
|  | Top third | 914 | 15 (1.6) | 0.99 (0.49 - 2.02) | 1.21 (0.54 - 2.75) | 1.47 (0.67 - 3.23) |
| Psychomotor change | Bottom third | 909 | 90 (9.9) | 1 [Reference] | 1 [Reference] | 1 [Reference] |
|  | Middle third | 908 | 118 (13.0) | 1.31 (1.01 - 1.70) | 1.30 (0.98 - 1.72) | 1.26 (0.95 - 1.67) |
|  | Top third | 914 | 121 (13.2) | 1.34 (1.03 - 1.73) | 1.33 (1.01 - 1.75) | 1.25 (0.93 - 1.67) |
| Change in appetite/weight | Bottom third | 905 | 225 (24.9) | 1 [Reference] | 1 [Reference] | 1 [Reference] |
|  | Middle third | 906 | 256 (28.3) | 1.14 (0.97 - 1.33) | 1.07 (0.91 - 1.25) | 1.05 (0.89 - 1.24) |
|  | Top third | 914 | 247 (27.0) | 1.09 (0.93 - 1.27) | 1.01 (0.86 - 1.19) | 0.98 (0.83 - 1.17) |
| Diurnal variation in mood | Bottom third | 909 | 44 (4.8) | 1 [Reference] | 1 [Reference] | 1 [Reference] |
|  | Middle third | 908 | 76 (8.4) | 1.73 (1.21 - 2.48) | 1.76 (1.19 - 2.62) | 1.92 (1.28 - 2.88) |
|  | Top third | 914 | 64 (7.0) | 1.45 (1.00 - 2.10) | 1.50 (0.99 - 2.27) | 1.75 (1.13 - 2.69) |
| Anhedonia | Bottom third | 905 | 147 (16.2) | 1 [Reference] | 1 [Reference] | 1 [Reference] |
|  | Middle third | 906 | 173 (19.1) | 1.18 (0.96 - 1.44) | 1.10 (0.89 - 1.37) | 1.09 (0.87 - 1.35) |
|  | Top third | 914 | 190 (20.8) | 1.28 (1.05 - 1.55) | 1.22 (0.99 - 1.51) | 1.19 (0.95 - 1.49) |
| Hopelessness | Bottom third | 905 | 249 (27.5) | 1 [Reference] | 1 [Reference] | 1 [Reference] |
|  | Middle third | 906 | 266 (29.4) | 1.07 (0.92 - 1.24) | 1.01 (0.87 - 1.18) | 1.01 (0.86 - 1.18) |
|  | Top third | 914 | 315 (34.5) | 1.25 (1.09 - 1.44) | 1.17 (1.01 - 1.36) | 1.17 (1.00 - 1.36) |
| Low self-esteem | Bottom third | 909 | 93 (10.2) | 1 [Reference] | 1 [Reference] | 1 [Reference] |
|  | Middle third | 908 | 126 (13.9) | 1.36 (1.05 - 1.74) | 1.26 (0.96 - 1.65) | 1.23 (0.94 - 1.62) |
|  | Top third | 914 | 135 (14.8) | 1.44 (1.13 - 1.85) | 1.32 (1.01 - 1.73) | 1.29 (0.98 - 1.70) |
| Self-blame | Bottom third | 909 | 82 (9.0) | 1 [Reference] | 1 [Reference] | 1 [Reference] |
|  | Middle third | 908 | 124 (13.7) | 1.51 (1.16 - 1.97) | 1.39 (1.04 - 1.85) | 1.34 (1.00 - 1.79) |
|  | Top third | 914 | 105 (11.5) | 1.27 (0.97 - 1.67) | 1.14 (0.85 - 1.54) | 1.08 (0.79 - 1.47) |
| Decreased libido | Bottom third | 909 | 33 (3.6) | 1 [Reference] | 1 [Reference] | 1 [Reference] |
|  | Middle third | 908 | 49 (5.4) | 1.49 (0.97 - 2.29) | 1.35 (0.85 - 2.14) | 1.39 (0.87 - 2.22) |
|  | Top third | 914 | 42 (4.6) | 1.27 (0.81 - 1.98) | 1.11 (0.68 - 1.81) | 1.22 (0.73 - 2.04) |
| Physical symptom score (top 25^th^ percentile) | Bottom third | 905 | 145 (16.0) | 1 [Reference] | 1 [Reference] | 1 [Reference] |
|  | Middle third | 906 | 169 (18.7) | 1.16 (0.95 - 1.42) | 1.09 (0.88 - 1.35) | 1.09 (0.88 - 1.36) |
|  | Top third | 914 | 177 (19.4) | 1.21 (0.99 - 1.48) | 1.08 (0.87 - 1.33) | 1.07 (0.85 - 1.34) |

^a^ Cut-off values for the top (66^th^ percentile) and bottom (33^rd^ percentile) levels of the distribution of serum IL-6 values in the total sample (those exhibiting and not exhibiting depressive symptoms) were 1.12 and 0.59 pg/mL, respectively

Abbreviations: BMI=Body Mass Index; CI=Confidence Interval; IL-6=Interleukin 6; No.=Number; RR=Risk Ratio

**Supplementary Table 3: Risk Ratios (95% CIs) for Specific Symptoms of Depression at Age 18 Years for Serum CRP Levels at Age 9 Years**

|  |  |  |  | **Risk Ratio (95% CI) for Depressive Symptom** | | |
| --- | --- | --- | --- | --- | --- | --- |
| **Depressive Symptom** | **CRP Group^a^** | **Sample** | **No. (%) with Symptom** | **Unadjusted** | **Adjusted for Age at Outcome, Sex, Father’s Occupation and Ethnicity** | **Further Adjusted for BMI and Self-reported Infections** |
| Fatigue | Bottom third | 988 | 271 (27.4) | 1 [Reference] | 1 [Reference] | 1 [Reference] |
|  | Middle third | 841 | 261 (31.0) | 1.13 (0.98 - 1.31) | 1.00 (0.86 - 1.16) | 0.99 (0.85 - 1.16) |
|  | Top third | 903 | 316 (35.0) | 1.28 (1.11 - 1.46) | 1.10 (0.95 - 1.27) | 1.06 (0.91 - 1.25) |
| Sleep disturbances | Bottom third | 988 | 204 (20.6) | 1 [Reference] | 1 [Reference] | 1 [Reference] |
|  | Middle third | 841 | 199 (23.7) | 1.15 (0.96 - 1.36) | 1.09 (0.90 - 1.31) | 1.06 (0.88 - 1.28) |
|  | Top third | 903 | 212 (23.4) | 1.14 (0.96 - 1.35) | 1.05 (0.87 - 1.27) | 0.96 (0.78 - 1.19) |
| Concentration difficulties | Bottom third | 988 | 111 (11.2) | 1 [Reference] | 1 [Reference] | 1 [Reference] |
|  | Middle third | 841 | 78 (9.3) | 0.83 (0.63 - 1.09) | 0.78 (0.59 - 1.05) | 0.78 (0.58 - 1.05) |
|  | Top third | 903 | 111 (12.3) | 1.09 (0.85 - 1.40) | 1.00 (0.77 - 1.31) | 1.01 (0.75 - 1.36) |
| Irritability | Bottom third | 988 | 227 (23.0) | 1 [Reference] | 1 [Reference] | 1 [Reference] |
|  | Middle third | 841 | 257 (30.6) | 1.33 (1.14 - 1.55) | 1.25 (1.05 - 1.47) | 1.25 (1.05 - 1.48) |
|  | Top third | 903 | 246 (27.2) | 1.19 (1.01 - 1.39) | 1.11 (0.93 - 1.31) | 1.08 (0.90 - 1.31) |
| Depressed/low mood | Bottom third | 988 | 93 (9.4) | 1 [Reference] | 1 [Reference] | 1 [Reference] |
|  | Middle third | 841 | 81 (9.6) | 1.02 (0.77 - 1.36) | 0.97 (0.71 - 1.33) | 0.96 (0.70 - 1.31) |
|  | Top third | 903 | 88 (9.7) | 1.04 (0.78 - 1.37) | 0.95 (0.70 - 1.29) | 0.92 (0.66 - 1.29) |
| Suicidality | Bottom third | 988 | 92 (9.3) | 1 [Reference] | 1 [Reference] | 1 [Reference] |
|  | Middle third | 841 | 100 (11.9) | 1.28 (0.98 - 1.67) | 1.17 (0.87 - 1.57) | 1.12 (0.84 - 1.51) |
|  | Top third | 903 | 87 (9.6) | 1.03 (0.78 - 1.37) | 0.91 (0.67 - 1.22) | 0.84 (0.60 - 1.17) |
| Worry | Bottom third | 988 | 188 (19.0) | 1 [Reference] | 1 [Reference] | 1 [Reference] |
|  | Middle third | 841 | 162 (19.3) | 1.01 (0.84 - 1.22) | 0.90 (0.74 - 1.11) | 0.91 (0.74 - 1.12) |
|  | Top third | 903 | 194 (21.5) | 1.13 (0.94 - 1.35) | 1.03 (0.85 - 1.24) | 0.98 (0.79 - 1.21) |
| Phobia | Bottom third | 988 | 53 (5.4) | 1 [Reference] | 1 [Reference] | 1 [Reference] |
|  | Middle third | 841 | 50 (5.9) | 1.11 (0.76 - 1.61) | 0.98 (0.66 - 1.46) | 0.96 (0.64 - 1.44) |
|  | Top third | 903 | 54 (6.0) | 1.11 (0.77 - 1.61) | 0.84 (0.56 - 1.25) | 0.79 (0.53 - 1.20) |
| Anxiety | Bottom third | 988 | 65 (6.6) | 1 [Reference] | 1 [Reference] | 1 [Reference] |
|  | Middle third | 841 | 60 (7.1) | 1.08 (0.77 - 1.52) | 0.97 (0.67 - 1.39) | 0.96 (0.67 - 1.39) |
|  | Top third | 903 | 70 (7.8) | 1.18 (0.85 - 1.63) | 1.02 (0.72 - 1.44) | 1.03 (0.71 - 1.50) |
| Panic | Bottom third | 992 | 16 (1.6) | 1 [Reference] | 1 [Reference] | 1 [Reference] |
|  | Middle third | 843 | 18 (2.1) | 1.32 (0.68 - 2.58) | 1.39 (0.65 - 2.94) | 1.44 (0.66 - 3.13) |
|  | Top third | 903 | 12 (1.3) | 0.82 (0.39 - 1.73) | 0.78 (0.34 - 1.80) | 0.94 (0.42 - 2.11) |
| Psychomotor change | Bottom third | 992 | 116 (11.7) | 1 [Reference] | 1 [Reference] | 1 [Reference] |
|  | Middle third | 843 | 104 (12.3) | 1.06 (0.82 - 1.35) | 0.97 (0.74 - 1.27) | 0.92 (0.70 - 1.21) |
|  | Top third | 903 | 110 (12.2) | 1.04 (0.82 - 1.33) | 0.95 (0.74 - 1.24) | 0.79 (0.59 - 1.06) |
| Change in appetite/weight | Bottom third | 988 | 236 (23.9) | 1 [Reference] | 1 [Reference] | 1 [Reference] |
|  | Middle third | 841 | 240 (28.5) | 1.19 (1.02 - 1.39) | 1.10 (0.93 - 1.30) | 1.09 (0.93 - 1.29) |
|  | Top third | 903 | 253 (28.0) | 1.17 (1.01 - 1.37) | 1.08 (0.92 - 1.27) | 1.07 (0.90 - 1.28) |
| Diurnal variation in mood | Bottom third | 992 | 72 (7.3) | 1 [Reference] | 1 [Reference] | 1 [Reference] |
|  | Middle third | 843 | 54 (6.4) | 0.88 (0.63 - 1.24) | 0.73 (0.51 - 1.07) | 0.76 (0.52 - 1.11) |
|  | Top third | 903 | 59 (6.5) | 0.90 (0.65 - 1.25) | 0.80 (0.56 - 1.14) | 0.86 (0.58 - 1.26) |
| Anhedonia | Bottom third | 988 | 182 (18.4) | 1 [Reference] | 1 [Reference] | 1 [Reference] |
|  | Middle third | 841 | 155 (18.4) | 1.00 (0.82 - 1.21) | 0.87 (0.70 - 1.07) | 0.85 (0.68 - 1.05) |
|  | Top third | 903 | 174 (19.3) | 1.05 (0.87 - 1.26) | 0.96 (0.78 - 1.17) | 0.87 (0.69 - 1.09) |
| Hopelessness | Bottom third | 988 | 285 (28.8) | 1 [Reference] | 1 [Reference] | 1 [Reference] |
|  | Middle third | 841 | 262 (31.2) | 1.08 (0.94 - 1.24) | 0.94 (0.81 - 1.10) | 0.94 (0.81 - 1.09) |
|  | Top third | 903 | 285 (31.6) | 1.09 (0.95 - 1.26) | 0.95 (0.82 - 1.10) | 0.92 (0.79 - 1.09) |
| Low self-esteem | Bottom third | 992 | 120 (12.1) | 1 [Reference] | 1 [Reference] | 1 [Reference] |
|  | Middle third | 843 | 118 (14.0) | 1.16 (0.91 - 1.47) | 1.02 (0.79 - 1.32) | 0.97 (0.75 - 1.25) |
|  | Top third | 903 | 116 (12.8) | 1.06 (0.84 - 1.35) | 0.93 (0.72 - 1.20) | 0.82 (0.62 - 1.09) |
| Self-blame | Bottom third | 992 | 108 (10.9) | 1 [Reference] | 1 [Reference] | 1 [Reference] |
|  | Middle third | 843 | 100 (11.9) | 1.09 (0.84 - 1.41) | 0.93 (0.70 - 1.22) | 0.88 (0.66 - 1.16) |
|  | Top third | 903 | 104 (11.5) | 1.06 (0.82 - 1.36) | 0.88 (0.67 - 1.15) | 0.76 (0.56 - 1.03) |
| Decreased libido | Bottom third | 992 | 44 (4.4) | 1 [Reference] | 1 [Reference] | 1 [Reference] |
|  | Middle third | 843 | 40 (4.7) | 1.07 (0.70 - 1.63) | 0.99 (0.62 - 1.55) | 1.02 (0.64 - 1.62) |
|  | Top third | 903 | 40 (4.4) | 1.00 (0.66 - 1.52) | 0.88 (0.57 - 1.38) | 0.92 (0.56 - 1.52) |
| Physical symptom score (top 25^th^ percentile) | Bottom third | 988 | 165 (16.7) | 1 [Reference] | 1 [Reference] | 1 [Reference] |
|  | Middle third | 841 | 146 (17.4) | 1.04 (0.85 - 1.27) | 0.91 (0.74 - 1.13) | 0.92 (0.74 - 1.14) |
|  | Top third | 903 | 181 (20.0) | 1.20 (0.99 - 1.45) | 0.98 (0.80 - 1.21) | 0.97 (0.77 - 1.22) |

^a^ Cut-off values for the top (66^th^ percentile) and bottom (33^rd^ percentile) thirds of the distribution of serum CRP levels in the total sample (those exhibiting and not exhibiting depressive symptoms) were 0.37 and 0.14 mg/L, respectively

Abbreviations: BMI=Body Mass Index; CI=Confidence Interval; CRP=C-Reactive Protein; No.=Number; RR=Risk Ratio

**Supplementary Table 4: Risk Ratios (95% CIs) for Specific Depressive Symptoms at Age 18 Years for Serum IL-6 Levels at Age 9 Years (Using IL-6 Quartiles)**

| **Depressive Symptom** | **IL-6 Group^a^** | **Sample** | **No. (%) with Symptom** | **RR (95% CI)** | |
| --- | --- | --- | --- | --- | --- |
|  |  |  |  | **Unadjusted** | **Adjusted for Age at Outcome, Sex, Father's Occupation, Ethnicity, BMI, Self-reported Infection** |
| Fatigue | Bottom quartile | 701 | 188 (26.8) | 1 [Reference] | 1 [Reference] |
|  | Second quartile | 660 | 181 (27.4) | 1.02 (0.86 - 1.22) | 0.98 (0.82 - 1.18) |
|  | Third quartile | 695 | 215 (30.9) | 1.15 (0.98 - 1.36) | 1.08 (0.91 - 1.29) |
|  | Top quartile | 670 | 264 (39.4) | 1.47 (1.26 - 1.71) | 1.34 (1.12 - 1.59) |
| Sleep disturbances | Bottom quartile | 701 | 133 (19.0) | 1 [Reference] | 1 [Reference] |
|  | Second quartile | 660 | 145 (22.0) | 1.16 (0.94 - 1.43) | 1.22 (0.97 - 1.53) |
|  | Third quartile | 695 | 173 (24.9) | 1.31 (1.07 - 1.60) | 1.23 (0.99 - 1.54) |
|  | Top quartile | 670 | 164 (24.5) | 1.29 (1.05 - 1.58) | 1.20 (0.95 - 1.52) |
| Concentration difficulties | Bottom quartile | 701 | 69 (9.8) | 1 [Reference] | 1 [Reference] |
|  | Second quartile | 660 | 70 (10.6) | 1.08 (0.79 - 1.48) | 1.03 (0.72 - 1.46) |
|  | Third quartile | 695 | 72 (10.4) | 1.05 (0.77 - 1.44) | 1.08 (0.76 - 1.52) |
|  | Top quartile | 670 | 89 (13.3) | 1.35 (1.00 - 1.81) | 1.45 (1.04 - 2.02) |
| Irritability | Bottom quartile | 701 | 169 (24.1) | 1 [Reference] | 1 [Reference] |
|  | Second quartile | 660 | 169 (25.6) | 1.06 (0.88 - 1.28) | 1.05 (0.86 - 1.28) |
|  | Third quartile | 695 | 194 (27.9) | 1.16 (0.97 - 1.38) | 1.04 (0.87 - 1.28) |
|  | Top quartile | 670 | 198 (30.0) | 1.23 (1.03 - 1.46) | 1.16 (0.95 - 1.42) |
| Depressed/low mood | Bottom quartile | 701 | 60 (8.6) | 1 [Reference] | 1 [Reference] |
|  | Second quartile | 660 | 61 (9.2) | 1.08 (0.77 - 1.52) | 1.11 (0.76 - 1.61) |
|  | Third quartile | 695 | 74 (10.6) | 1.24 (0.90 - 1.72) | 1.10 (0.76 - 1.59) |
|  | Top quartile | 670 | 67 (10.0) | 1.17 (0.84 - 1.63) | 1.11 (0.76 - 1.63) |
| Suicidality | Bottom quartile | 701 | 67 (9.6) | 1 [Reference] | 1 [Reference] |
|  | Second quartile | 660 | 62 (9.4) | 0.98 (0.71 - 1.37) | 0.98 (0.68 - 1.41) |
|  | Third quartile | 695 | 66 (9.5) | 0.99 (0.72 - 1.37) | 0.85 (0.59 - 1.24) |
|  | Top quartile | 670 | 84 (12.5) | 1.31 (0.97 - 1.78) | 1.37 (0.97 - 1.93) |
| Worry | Bottom quartile | 701 | 129 (18.4) | 1 [Reference] | 1 [Reference] |
|  | Second quartile | 660 | 138 (20.9) | 1.14 (0.92 - 1.41) | 1.12 (0.89 - 1.41) |
|  | Third quartile | 695 | 122 (17.6) | 0.95 (0.76 - 1.19) | 0.87 (0.68 - 1.12) |
|  | Top quartile | 670 | 155 (23.1) | 1.26 (1.02 - 1.55) | 1.12 (0.88 - 1.42) |
| Phobia | Bottom quartile | 701 | 29 (4.1) | 1 [Reference] | 1 [Reference] |
|  | Second quartile | 660 | 38 (5.8) | 1.39 (0.87 - 2.23) | 1.43 (0.86 - 2.38) |
|  | Third quartile | 695 | 46 (6.6) | 1.60 (1.02 - 2.52) | 1.45 (0.89 - 2.37) |
|  | Top quartile | 670 | 44 (6.6) | 1.59 (1.01 - 2.51) | 1.40 (0.84 - 2.34) |
| Anxiety | Bottom quartile | 701 | 46 (6.6) | 1 [Reference] | 1 [Reference] |
|  | Second quartile | 660 | 46 (7.0) | 1.06 (0.72 - 1.58) | 1.11 (0.73 - 1.68) |
|  | Third quartile | 695 | 44 (6.3) | 0.96 (0.65 - 1.44) | 0.88 (0.57 - 1.37) |
|  | Top quartile | 670 | 59 (8.8) | 1.34 (0.93 - 1.94) | 1.34 (0.88 - 2.02) |
| Panic | Bottom quartile | 705 | 13 (1.8) | 1 [Reference] | 1 [Reference] |
|  | Second quartile | 661 | 9 (1.4) | 0.74 (0.32 - 1.72) | 1.12 (0.42 - 2.97) |
|  | Third quartile | 696 | 13 (1.9) | 1.01 (0.47 - 2.17) | 1.14 (0.44 - 2.92) |
|  | Top quartile | 670 | 11 (1.6) | 0.89 (0.40 - 1.97) | 1.65 (0.67 - 4.07) |
| Psychomotor change | Bottom quartile | 705 | 67 (9.5) | 1 [Reference] | 1 [Reference] |
|  | Second quartile | 661 | 86 (13.0) | 1.37 (1.01 - 1.85) | 1.44 (1.04 - 2.01) |
|  | Third quartile | 696 | 86 (12.4) | 1.30 (0.96 - 1.76) | 1.22 (0.87 - 1.71) |
|  | Top quartile | 670 | 91 (13.6) | 1.43 (1.06 - 1.92) | 1.40 (1.00 - 1.96) |
| Change in appetite/weight | Bottom quartile | 701 | 170 (24.3) | 1 [Reference] | 1 [Reference] |
|  | Second quartile | 660 | 185 (28.0) | 1.16 (0.97 - 1.38) | 1.12 (0.93 - 1.35) |
|  | Third quartile | 695 | 200 (28.8) | 1.19 (1.00 - 1.41) | 1.01 (0.84 - 1.22) |
|  | Top quartile | 670 | 173 (25.8) | 1.06 (0.89 - 1.28) | 0.98 (0.80 - 1.20) |
| Diurnal variation in mood | Bottom quartile | 705 | 35 (5.0) | 1 [Reference] | 1 [Reference] |
|  | Second quartile | 661 | 52 (7.9) | 1.58 (1.05 - 2.40) | 1.96 (1.23 - 3.13) |
|  | Third quartile | 696 | 48 (6.9) | 1.39 (0.91 - 2.12) | 1.66 (1.02 - 2.69) |
|  | Top quartile | 670 | 50 (7.5) | 1.50 (0.99 - 2.29) | 1.90 (1.16 - 3.10) |
| Anhedonia | Bottom quartile | 701 | 115 (16.4) | 1 [Reference] | 1 [Reference] |
|  | Second quartile | 660 | 127 (19.2) | 1.17 (0.93 - 1.47) | 1.16 (0.91 - 1.49) |
|  | Third quartile | 695 | 125 (18.0) | 1.10 (0.87 - 1.38) | 1.02 (0.79 - 1.31) |
|  | Top quartile | 670 | 144 (21.5) | 1.31 (1.05 - 1.63) | 1.27 (0.98 - 1.63) |
| Hopelessness | Bottom quartile | 701 | 194 (27.7) | 1 [Reference] | 1 [Reference] |
|  | Second quartile | 660 | 183 (27.7) | 1.00 (0.84 - 1.19) | 1.02 (0.85 - 1.22) |
|  | Third quartile | 695 | 218 (31.4) | 1.13 (0.96 - 1.33) | 1.04 (0.87 - 1.24) |
|  | Top quartile | 670 | 236 (35.2) | 1.27 (1.09 - 1.49) | 1.26 (1.06 - 1.50) |
| Low self-esteem | Bottom quartile | 705 | 71 (10.1) | 1 [Reference] | 1 [Reference] |
|  | Second quartile | 661 | 83 (12.6) | 1.25 (0.92 - 1.68) | 1.20 (0.87 - 1.65) |
|  | Third quartile | 696 | 97 (13.9) | 1.38 (1.04 - 1.85) | 1.14 (0.83 - 1.56) |
|  | Top quartile | 670 | 103 (15.4) | 1.53 (1.15 - 2.03) | 1.37 (1.00 - 1.87) |
| Self-blame | Bottom quartile | 705 | 61 (8.7) | 1 [Reference] | 1 [Reference] |
|  | Second quartile | 661 | 83 (12.6) | 1.45 (1.06 - 1.98) | 1.45 (1.04 - 2.04) |
|  | Third quartile | 696 | 87 (12.5) | 1.44 (1.06 - 1.97) | 1.21 (0.85 - 1.71) |
|  | Top quartile | 670 | 81 (12.1) | 1.40 (1.02 - 1.91) | 1.21 (0.84 - 1.73) |
| Decreased libido | Bottom quartile | 705 | 28 (4.0) | 1 [Reference] | 1 [Reference] |
|  | Second quartile | 661 | 28 (4.2) | 1.07 (0.64 - 1.78) | 1.25 (0.73 - 2.14) |
|  | Third quartile | 696 | 37 (5.3) | 1.34 (0.83 - 2.16) | 1.21 (0.70 - 2.07) |
|  | Top quartile | 670 | 31 (4.6) | 1.16 (0.71 - 1.92) | 1.17 (0.65 - 2.11) |
| Physical symptom score (top 25^th^ percentile) | Bottom quartile | 701 | 111 (15.8) | 1 [Reference] | 1 [Reference] |
|  | Second quartile | 660 | 123 (18.6) | 1.18 (0.93 - 1.49) | 1.12 (0.87 - 1.44) |
|  | Third quartile | 695 | 133 (19.1) | 1.21 (0.96 - 1.52) | 1.04 (0.81 - 1.33) |
|  | Top quartile | 670 | 125 (18.7) | 1.18 (0.93 - 1.49) | 1.03 (0.79 - 1.34) |

^a^ The cut-off values for the 75^th^, 50^th^ and 25^th^ quartiles of the distribution of serum CRP levels in the total sample (those exhibiting and not exhibiting depressive symptoms) were 1.42, 0.81 and 0.41 pg/mL, respectively

**Supplementary Table 5: Comparison of Baseline Characteristics Between the IL-6 Analytic (n = 2731) and Missing Data (n = 2343) Samples at Age 9 Years**

|  | **IL-6 Analytic vs. Missing Data Samples at Age 9 Years** | |  |
| --- | --- | --- | --- |
| **Characteristic** | **IL-6 Analytic Sample^a^** | **Missing Data Sample^b^** | ***P*-value^c^** |
| Total no. of participants | 2731 | 2343 |  |
| Log transformed serum IL-6 levels (log pg/ml) at age 9 years, mean (SD)^d^ | -0.16 (0.88) | -0.15 (0.85) | 0.48 |
| Serum IL-6 levels (pg/ml) at age 9 years, median (IQR)^e^ | 0.81 (0.49 - 1.41) | 0.81 (0.51 - 1.44) | 0.62 |
| Age (months) at outcome, mean (SD) | 212.9 (4.4) | 215.4 (7.2) | <0.001 |
| Male sex, no. (%) | 1240 (45.4) | 1321 (56.4) | <0.001 |
| BMI at age 9 years, median (IQR)^f^ | 21.8 (20.0 - 24.1) | 22.0 (20.1 - 24.6) | 0.24 |
| British white ethnicity, no. (%)^g^ | 2504 (98.0) | 2061 (98.3) | 0.41 |
| Paternal social class, non-manual, no. (%)^h^ | 1579 (66.0) | 1072 (56.2) | <0.001 |
| Infection present at age 9 years, no (%)^i^ | 269 (9.9) | 211 (9.0) | 0.31 |

**^a^** The analytic sample was comprised of participants who had data on both their serum IL-6 levels at age 9 years and their follow-up assessment at age 18 years

^b^ The missing data sample constituted of participants who had data on their serum IL-6 levels at age 9 years but were missing data from their follow-up assessment at age 18 years

^c^ Welch's unpaired t-test was used to assess the mean log-transformed serum IL-6 levels at age 9 years and ages between the two samples; the Mann-Whitney U/Wilcoxon rank-sum test was used to assess median serum IL-6 levels and BMI at age 9 years between the two samples; the chi-squared test for proportions was used to test equal proportions of categorical characteristics (sex, ethnicity, paternal social class and infection status at age 9 years) between the two samples

^d^ Serum IL-6 levels at age 9 years were log-transformed to yield a normal distribution; the mean and SD of the log-transformed distribution were then reported as measures of location and distribution

^e^ The median value and IQR were reported as alternative measures of location and distribution of the untransformed serum IL-6 levels at age 9 years

^f^ The median value and IQR for BMI at age 9 years were reported due to a lack of normality in the distribution of BMI values

^g^ Data on ethnicity was available for only 4651 participants across the two samples; the denominators for the analytic and missing data samples were 2555 and 2096, respectively

^h^ Data on paternal social class was available for only 4300 participants across the two samples; the denominators for the analytic and missing data samples were 2391 and 1909, respectively

^i^ Data on infection status age 9 years was available for only 5065 participants across the two samples; the denominators for the analytic and missing data samples were 2726 and 2339, respectively

Abbreviations: BMI=Body Mass Index; IQR=Interquartile Range; SD=Standard Deviation

**Supplementary Table 6: Risk Ratios (95% CIs) for Specific Symptoms of Depression at Age 18 Years for Serum CRP Levels at Age 9 Years After Excluding Participants (n = 60) with CRP >10 mg/L**

|  |  |  |  | **Risk Ratio (95% CI) for Depressive Symptom** | | |
| --- | --- | --- | --- | --- | --- | --- |
| **Depressive Symptom** | **CRP Group^a^** | **Sample** | **No. (%) with Symptom** | **Unadjusted** | **Adjusted for Age at Outcome, Sex, Father’s Occupation and Ethnicity** | **Further Adjusted for BMI and Self-reported Infections** |
| Fatigue | Bottom third | 988 | 271 (27.4) | 1 [Reference] | 1 [Reference] | 1 [Reference] |
|  | Middle third | 841 | 261 (31.0) | 1.13 (0.98 - 1.31) | 1.00 (0.85 - 1.16) | 0.99 (0.85 - 1.16) |
|  | Top third | 874 | 302 (34.6) | 1.26 (1.10 - 1.44) | 1.08 (0.94 - 1.25) | 1.05 (0.90 - 1.24) |
| Sleep disturbances | Bottom third | 988 | 204 (20.6) | 1 [Reference] | 1 [Reference] | 1 [Reference] |
|  | Middle third | 841 | 199 (23.7) | 1.15 (0.96 - 1.36) | 1.09 (0.90 - 1.31) | 1.06 (0.88 - 1.29) |
|  | Top third | 874 | 205 (23.5) | 1.14 (0.96 - 1.35) | 1.05 (0.86 - 1.27) | 0.96 (0.78 - 1.19) |
| Concentration difficulties | Bottom third | 988 | 111 (11.2) | 1 [Reference] | 1 [Reference] | 1 [Reference] |
|  | Middle third | 841 | 78 (9.3) | 0.83 (0.63 - 1.09) | 0.78 (0.59 - 1.05) | 0.78 (0.58 - 1.05) |
|  | Top third | 874 | 107 (12.2) | 1.09 (0.85 - 1.40) | 0.99 (0.76 - 1.30) | 0.99 (0.73 - 1.34) |
| Diurnal variation in mood | Bottom third | 992 | 72 (7.3) | 1 [Reference] | 1 [Reference] | 1 [Reference] |
|  | Middle third | 843 | 54 (6.4) | 0.88 (0.63 - 1.24) | 0.74 (0.51 - 1.07) | 0.76 (0.53 - 1.11) |
|  | Top third | 874 | 59 (6.8) | 0.93 (0.67 - 1.30) | 0.82 (0.58 - 1.17) | 0.88 (0.60 - 1.29) |

^a^ Cut-off values for the top (66^th^ percentile) and bottom (33^rd^ percentile) thirds of the distribution of serum CRP levels in the total sample (those exhibiting and not exhibiting depressive symptoms) were 0.37 and 0.14 mg/L, respectively (after excluding 60 participants with CRP >10 mg/L from the CRP analytic sample)

Abbreviations: BMI=Body Mass Index; CI=Confidence Interval; CRP=C-Reactive Protein; No.=Number; RR=Risk Ratio
